# Generation of Friedreich’s ataxia induced pluripotent stem cells carrying the FXN c.165 + 5G>C splicing mutation

**DOI:** 10.1016/j.scr.2026.103966

**Published:** 2026-03-16

**Authors:** Pouiré Yameogo, Brandon J. Gerhart, Monica F. Sentmanat, Amber Neilson, Xiaoxia Cui, Mayank Verma, David R. Lynch, Jill S. Napierala, Marek Napierala

**Affiliations:** a Department of Neurology, O’Donnell Brain Institute, University of Texas Southwestern Medical Center, 5323 Harry Hines Blvd., Dallas, TX 75390, USA; b Genome Engineering & Stem Cell Center (GESC@MGI), Department of Genetics, Washington University School of Medicine in St. Louis, St. Louis, MO 63108, USA; c Department of Pediatrics, Division of Pediatric Neurology, O’Donnell Brain Institute, University of Texas Southwestern Medical Center, 5323 Harry Hines Blvd., Dallas, TX 75390, USA; d Department of Pediatrics and Neurology, The Children’s Hospital of Philadelphia, Philadelphia, PA 19104, USA

## Abstract

Friedreich’s ataxia (FRDA) is a multisystem, autosomal recessive disease caused by biallelic expansion of GAA repeats in intron 1 of the frataxin gene (FXN). While ~96% of FRDA patients carry expanded GAA repeats on both FXN alleles, ~4% are compound heterozygous with expanded GAA repeats on one allele and another mutation on the second allele. We generated induced pluripotent stem cells from blood lymphocytes from a FRDA patient carrying the FXN c.165 + 5G > C point mutation, which interferes with canonical splicing of intron 1 of the FXN gene. These cells allow for development of therapeutic approaches that target splicing defect in FRDA.

**1.** Resource table

Unique stem cell line identifier	UTSWi004-A; https://hpscreg.eu/cell-line/UTSWi004-A
Alternative name(s) of stem cell line	N/A
Institution	University of Texas Southwestern Medical Center
Contact information of distributor	*Marek Napierala*,
Type of cell line	iPSC
Origin	human
Additional origin info	*Age: N/A* Sex: male *Ethnicity if known: N/A*
Cell Source	Lymphocyte
Method of reprogramming	Sendai virus
Associated disease	Friedreich’s Ataxia
Gene/locus	N/A
Method of modification	N/A
Gene correction	NO
Name of transgene or resistance	N/A
Inducible/constitutive system	N/A
Date archived/stock date	N/A
Cell line repository/bank	Friedreich’s Ataxia Cell Line Repository (FACLR), https://labs.utsouthwestern.edu/napierala-lab/cell-line-repository
Ethical approval	Children’s Hospital of Philadelphia IRB 10–007864 and UT Southwestern Medical Center IRB #STU-2023–0478

## 2. Resource utility

Friedreich’s ataxia is a multisystem, autosomal recessive disease caused by mutations in the frataxin gene (FXN). The iPSC line carrying FXN c.165+5G>C mutation in the intron 1 described in this resource will enable the testing of therapeutic strategies in disease-relevant differentiated cells and 3D cellular models of the disease (see Table 1).

## 3. Resource details

Friedreich’s ataxia (FRDA) is a rare multisystem disease predominantly caused by biallelic expansion of Alu-derived GAA trinucleotide repeats (Campuzano, 1996; Clark, 2004). While numerous point mutations have been reported in the literature, FRDA patients harboring mutations other than GAA repeat expansions are rare (Shen et al., 2024). Individual properties of point mutations allow for the development of personalized therapeutic approaches. Intronic mutations resulting in aberrant splicing are of special interest, due to a large body of preclinical and clinical studies that resulted in approvals of specific therapies targeting splicing defects (Lauffer, 2024; McCormack, 2000). The FXN c.165+5G>C mutation has been shown to affect canonical splicing of FXN, which can be improved by treatment with antisense oligonucleotides (ASO) (Yameogo, 2025).

**Fig. 1.**
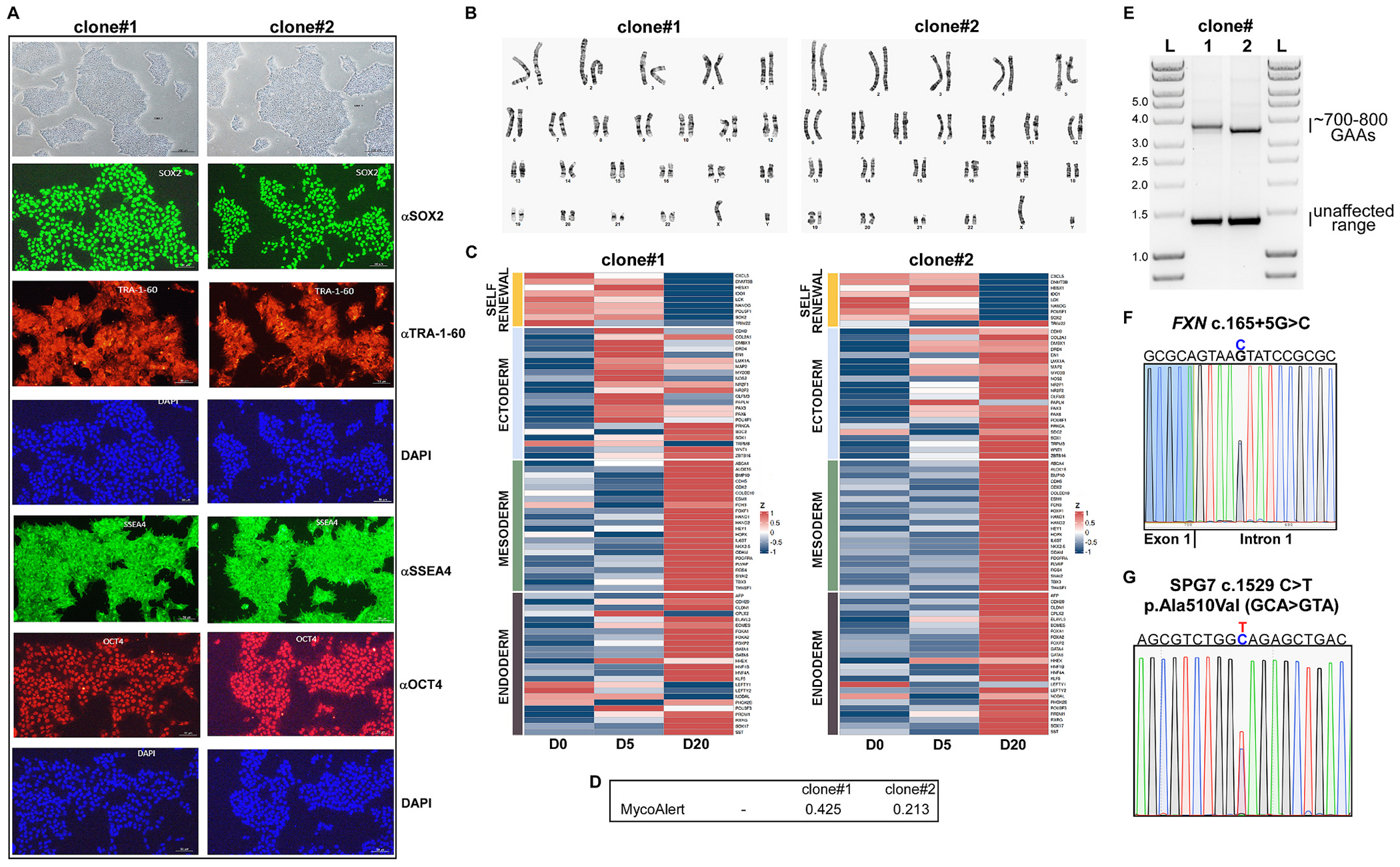


The FXN c.165+5G>C FRDA iPSCs were generated from a peripheral blood sample (CHOP IRB 10–007864) using four transcription factors delivered via Sendai virus transduction. Two independent clones (clone#1 and clone#2) were isolated, expanded, and characterized. Both clones demonstrated human pluripotent stem cell morphology (Fig. 1A) and were immunopositive for nuclear (SOX2, OCT4) and surface (TRA-1–60, SSEA4) pluripotency markers. No karyotype abnormalities were detected using G-banding analysis (Fig. 1B). The shorttandem repeat (STR) profiling of 16 loci confirmed a genetic match between parental peripheral blood mononuclear cells (PBMCs) and iPSCs.

The FXN c.165+5G>C FRDA iPSCs clones were evaluated for expression of key lineage-specific and pluripotency markers based on established gene sets using RNA sequencing followed by lineage ScoreCard analysis of the expression of 77 transcripts (9 self-renewal specific, 22 ectoderm, 22 mesoderm, and 24 endoderm specific; Fig. 1C). The iPSCs were spontaneously differentiated followed by RNA isolation and transcriptome sequencing at day 0 (D0, iPSCs), day 5 (D5), and day 20 (D20) post-differentiation. Both clones passed the analysis criteria (≥ 70% of expressed markers trend in the expected direction and ≤ 30% show strong wrong-direction changes) for initial pluripotency (D0) and trilineage differentiation capability. Both iPSC clones tested negative for mycoplasma (Fig. 1D).

To confirm the molecular phenotype of the iPSCs, the size of the GAA tract in intron 1 of the FXN gene was analyzed using long-range PCR. Results demonstrated that one of the FXN alleles harbors an expanded GAA tract of ~700 – 800 repeats (pathogenic repeats are greater than 50 units), while the second allele contained a short non-pathogenic GAA tract (Fig. 1E). To confirm the presence of the FXN c.165+5G>C point mutation, a PCR fragment spanning the distal sequence of exon 1 and the proximal sequence of intron 1 was amplified and subjected to Sanger sequencing (Fig. 1F). This FRDA patient was also a carrier of the c.1529C>T point mutation in exon 11 in the Spastic Paraplegia type 7 (SPG7) gene as confirmed by PCR and sequencing (Fig. 1G). The FXN c.165+5G>C FRDA iPSCs are available from UT Southwestern Medical Center Friedreich’s Ataxia Cell Line Repository.

## 4. Materials and methods

### 4.1. Reprogramming of blood lymphocytes to iPSCs

Human peripheral blood mononuclear cells (PBMCs) were reprogrammed using the CytoTune iPS 2.0 Sendai Reprogramming Kit (Thermo Fisher Scientific, Cat. No. A16517), according to the manufacturer’s instructions with minor modifications. Briefly, PBMCs were plated at a density of 5 × 105 cells/mL in one well of a 24-well plate in complete PBMC medium (Day −4). From Day −3 to Day −1, half of the medium was replaced daily with fresh complete PBMC medium. On Day 0, cells were transduced with CytoTune 2.0 Sendai viral vectors at the multiplicity of infection (MOI) specified for the individual kit lot. Transduction was performed by centrifugation at 1,000 × g for 15 min. Following centrifugation, cells and virus-containing medium were transferred to one well of an uncoated 6-well plate and incubated overnight at 37 °C. On Day 1, the medium was replaced with fresh complete PBMC medium to remove residual Sendai virus. On Day 3, the medium was changed to a 1:1 mixture of PBMC medium and Essential 8 (E8/eTeSR) medium. On Day 4, cells were plated onto Matrigel-coated culture dishes and maintained in complete eTeSR medium. From Day 5 to Day 28, cultures were fed with fresh complete eTeSR medium every other day and monitored for the appearance of induced pluripotent stem cell (iPSC) colonies. Emerging colonies with undifferentiated morphology were manually picked and transferred onto fresh Matrigel-coated dishes for expansion. Unless otherwise indicated all analyses were performed on passage 2–5 iPSCs.

### 4.2. Immunostaining for pluripotency markers

Pluripotency marker expression was assessed using the PSC Immunocytochemistry Kit (Thermo Fisher Scientific, Cat. No. A24881) following the manufacturer’s protocol. Cells were fixed and per-meabilized using reagents supplied in the kit, followed by incubation in blocking solution for 30 min at room temperature. Cells were then incubated overnight at 4 °C with the primary antibodies provided in the kit (anti-SSEA4, anti-OCT4, anti-SOX2, and anti-TRA-1–60). After primary antibody incubation, cells were washed three times with 1× wash buffer and incubated with blocking solution containing the appropriate fluorophore-conjugated secondary antibodies for 1 h at room temperature. Cells were washed three additional times with 1× wash buffer. Nuclear staining was performed during the final wash using NucBlue Fixed Cell Stain (DAPI; 2 drops/mL) for 5 min. Cells were imaged using fluorescence microscopy.

### 4.3. Karyotype analysis

Karyotype analyses using G-banding was performed and analyzed at Clinical Genomics Laboratory at University of Washington School of Medicine, St. Louis, MO, USA.

### 4.4. STR profiling

A set of 16 STR loci was included in the analyses. STR loci were amplified for next-generation sequencing using a two-step PCR strategy as previously described (Chen, 2023). In the first PCR (PCR1), individual or multiplexed STR loci were amplified using Platinum SuperFi II PCR Master Mix (ThermoFisher, Cat. No. 12369250) using primers with universal tails and partial Illumina adapters. A second PCR (PCR2) was performed using PCR1 amplicons as input to add full Illumina adapters and unique dual indices. Final libraries were purified using SPRI beads and sequenced on an Illumina MiSeq (2×250 bp) using standard Illumina workflows. Reads were demultiplexed and FASTQ files analyzed using the STRight python script (Chen, 2023).

### 4.5. Mycoplasma testing

Testing was performed on 48h-old iPSC media using the MycoAlert Mycoplasma Detection Assay (Lonza, LT07-318) according to the manufacturer’s protocol. A luminescence ratio <0.9 indicates a sample free of mycoplasma.

### 4.6. Trilineage differentiation potential determination

Complete KnockOut Serum Replacement (KSR) embryoid body (EB) medium was prepared by combining KnockOut DMEM/F12 (ThermoFisher Scientific, Cat. No. 12660–012), 20% KnockOut Serum Replacement (Cat. No. 10828–028), 2 mM GlutaMAX (Cat. No. 35050–061), and 0.1 mM Non-Essential Amino Acids (Cat. No. 11140–076). On Day 0, iPSCs maintained in eTeSR medium were assessed for morphology and quality. On Day 1, cells were dissociated using Accutase, counted, and resuspended at 4–6 × 10^6^ cells in 10 mL eTeSR supplemented with 10 μM ROCK inhibitor (Y-27632). Aliquots of 100 μL cell suspension were plated into each well of a 96-well V-bottom plate and centrifuged at 100 × g for 3 min to promote aggregation. From Day 2 to Day 5, half of the medium was replaced daily with fresh eTeSR containing 10 μM ROCK inhibitor. On Day 5, EBs were transferred to Matrigel-coated 6-well plates (32 EBs per well) in complete KSR EB medium. From Day 6 to Day 21, medium was replaced every other day with fresh EB medium (3 mL per well). EBs were cultured until attachment, flattening, and spontaneous differentiation consistent with formation of derivatives of the three germ layers was observed. RNA was isolated at D0 (iPSCs), D5 and D20 of differentiation and gene expression was analyzed by RNA sequencing. Raw gene-level counts were imported and normalized using DESeq2 size factors and gene expression data were imported as log2 counts per million (log2CPM). Curated gene sets representing pluripotency (self-renewal) and germ layer lineages (ectoderm, mesoderm, endoderm) were defined based on (Bock, 2011). For each gene, log2CPM values at D0, D5, and D20 were used to perform gene-wise z-score normalization across timepoints within the clone. Heatmaps were generated using the ComplexHeatmap package (v2.24.1), with rows grouped by lineage and columns ordered by differentiation timepoint. Pass/Fail Scorecard Generation: For each lineage and pluripotency gene set, marker genes were evaluated based on their direction of change between D0 and D20. Genes were considered expressed if they exceeded a minimum normalized count threshold at either D0 or D20. A gene was counted as trending in the expected direction if its expression increased from D0 to D20 for lineage markers (ectoderm, endoderm, mesoderm) or decreased for pluripotency markers. A gene was counted as strongly wrong-direction only if it showed a ≥ 2-fold change (|log2FC| ≥ 1) in the opposite direction. The iPSCs pass trilineage differentiation testing if ≥ 70% of expressed markers trend in the expected direction and ≤ 30% show strong wrong-direction changes.

### 4.7. Long-range GAA PCR

Amplification of the expanded GAA repeat tract was performed using primers listed in Table 2 as we described in (Long, 2017).

### 4.8. Detection of point mutations

To detect FXN and SPG7 point mutation in the iPSCs, a fragment of the respective gene was amplified using CloneAmp HiFi PCR Premix (Takara Bio Inc., Cat# 639298) using primers indicated in Table 2. The reaction conditions were the same for both genes: an initial denaturation of 30 s at 98°C, 34 cycles of 10 s at 98°C, 15 s at 60°C, and 10 s at 72°C followed by final elongation step at 72°C for 5 min. PCR products were analyzed on 1.5% agarose gels, purified using a PCR Purification Kit (Qiagen, Cat# 28104) and sequenced.

## Figures and Tables

**Table 1:** Characterization and validation of hiPSCs.

Classification	Test	Result	Data
**Morphology**	Photography bright field	Normal hiPSC morphology	Fig. 1 A
**Phenotype**	Qualitative Analysis by immunocytochemisty	hiPSCs express SOX2, TRA-1–60, SSEA4 and OCT4 markers	Fig. 1 A
**Genotype**	Karyotype (G-banding)	46,XY, no abnormalities detected	Fig. 1B
**Identity**	Microsatellite PCR (mPCR)	Not performed	N/A
	STR analysis	Tested for 16 markers, matched	submitted in archive with journal
**Mutation analysis**	PCR and sequencing	PCR: GAA repeat expansion; Sequencing: heterozygous for FXN c.165 + 5 G > C Sequencing: heterozygous for SPG7 c1529 C > T	Fig. 1E Fig. 1F Fig. 1G
**Microbiology and virology**	Mycoplasma by MycoAlert	Negative	Fig. 1 D
**Differentiation potential**	ScoreCard analysis using RNA sequencing of embryoid bodies	Expression of 9 self-renewal specific, 22 ectoderm, 22 mesoderm, and 24 endoderm specific confirmed trilineage differentiation potential	Fig. 1C
**Donor screening (OPTIONAL)**	HIV 1 + 2 Hepatitis B, Hepatitis C	N/A	N/A
**Genotype**	Blood group genotyping	N/A	N/A
**additional info (OPTIONAL)**	HLA tissue typing	N/A	N/A

**Table 2:** Reagents details.

Antibodies used for immunocytochemistry			
	Antibody	Dilution	Company Cat # and RRID
Pluripotency Markers	Rabbit anti- OCT4	1:200	Pluripotent Stem Cell 4-Marker Immunocytochemistry Kit, Thermo Fisher Scientific, Cat. No. A24881 and A24867
	Mouse anti-SSEA4	1:100	Pluripotent Stem Cell 4-Marker Immunocytochemistry Kit, Thermo Fisher Scientific, Cat. No. A24881 and A24866
	Rat anti-SOX2	1:100	Pluripotent Stem Cell 4-Marker Immunocytochemistry Kit, Thermo Fisher Scientific, Cat. No. A24881 and A24759
	Mouse anti-TRA-1–60	1:100	Pluripotent Stem Cell 4-Marker Immunocytochemistry Kit, Thermo Fisher Scientific, Cat. No. A24881 and A24868
Secondary antibodies	Alexa Fluor^™^ 555 donkey anti-rabbit; for anti-OCT4	1:250	Pluripotent Stem Cell 4-Marker Immunocytochemistry Kit, Thermo Fisher Scientific, Cat. No. A24881 and A24869
	Alexa Fluor^™^ 488 goat anti-mouse for anti-SSEA4	1:250	Pluripotent Stem Cell 4-Marker Immunocytochemistry Kit, Thermo Fisher Scientific, Cat. No. A24881 and A24877
	Alexa Fluor^™^ 488 donkey anti-rat for anti-SOX2	1:250	Pluripotent Stem Cell 4-Marker Immunocytochemistry Kit, Thermo Fisher Scientific, Cat. No. A24881 and A24876
	Alexa Fluor^™^ 594 goat anti-mouse; for anti-TRA-1–60	1:250	Pluripotent Stem Cell 4-Marker Immunocytochemistry Kit, Thermo Fisher Scientific, Cat. No. A24881 and A24872
Primers			
	Target	Forward/Reverse primer (5′-3′)	
Targeted mutation analysis	*FXN* intron 1 GAA repeat PCR	Fwd: GGAGGGAACCGTCTGGGCAAAGG Rev: CAATCCAGGACAGTCAGGGCTTT	
Targeted mutation analysis and sequencing	*FXN* c.165 + 5 G > C mutation	Fwd: GTCAGGGGTCCTGGTTGCAC Rev: TCACACCAGGTCCGCAAAAT	
Targeted mutation analysis and sequencing	*SPG7* c.1529C > T mutation	Fwd: CGCACCTGTGGCAGTAACTA Rev: CTCTGGGTCTGACGGGAAAC	
